# The effect of antibiotic usage on resistance in humans and food-producing animals: a longitudinal, One Health analysis using European data

**DOI:** 10.3389/fpubh.2023.1170426

**Published:** 2023-06-15

**Authors:** Sakib Rahman, Aidan Hollis

**Affiliations:** Department of Economics, University of Calgary, Calgary, AB, Canada

**Keywords:** antibiotics, resistance, Europe, causality, interspecies effect, One Health

## Abstract

This paper estimates the effect of antibiotic usage in humans and food-producing animals on the prevalence of resistance in zoonotic bacteria in both humans and animals. Using comprehensive longitudinal data from annual surveillance reports on resistance and usage in Europe, we find that antibiotic usage in food-producing animals and antibiotic usage in humans are independently and causally related to the prevalence of resistance in both humans and animals. The study considers simultaneous and total usage of antibiotics in humans and food-producing animals to identify the marginal effects and joint effects of usage on resistance of both groups. By employing lagged-dependent variable and fixed-effects specifications, we provide a lower and an upper bound on the effects on resistance. The paper also contributes to the scant literature on how antibiotic use in humans is related to resistance in other animals.

## 1. Introduction

Antimicrobial resistance not only poses a growing public health threat to humans but also risks animal health and production (1–3). Antimicrobial agents used in human medicine often belong to the same classes as those used in food-producing animals and many antimicrobials are used in both humans and animals (4, 5). Given the overlap of antibiotics used in these populations, there is a growing concern that the extensive usage of antibiotics in one population could contribute to the development of resistance to antibiotics commonly used in the other (6). The extent of this well-documented biological relationship is, however, not well characterized at an ecological level, i.e., across time and space, and a consensus is yet to be reached on the empirical connection between usage and resistance within and across humans and food-producing animals. It is crucial to approach this relationship from a One Health perspective since policies, regulations and stewardship in one sector can affect other interrelated sectors.

While usage of antimicrobials is a primary contributor to resistance, evidence suggests there are socioeconomic, institutional and environmental factors which also play a role (7–11). The issue also lies at the intersection of various scientific, technical, behavioral, ecological, and economic disciplines which renders forming informed approaches to alleviate the problem even more complex. Researchers from various fields are studying to gain a better understanding of antimicrobial resistance but the diverse perspectives and innate complexities involved results in a lack of consensus and at times conflicting findings (12).

**Figure 1:**
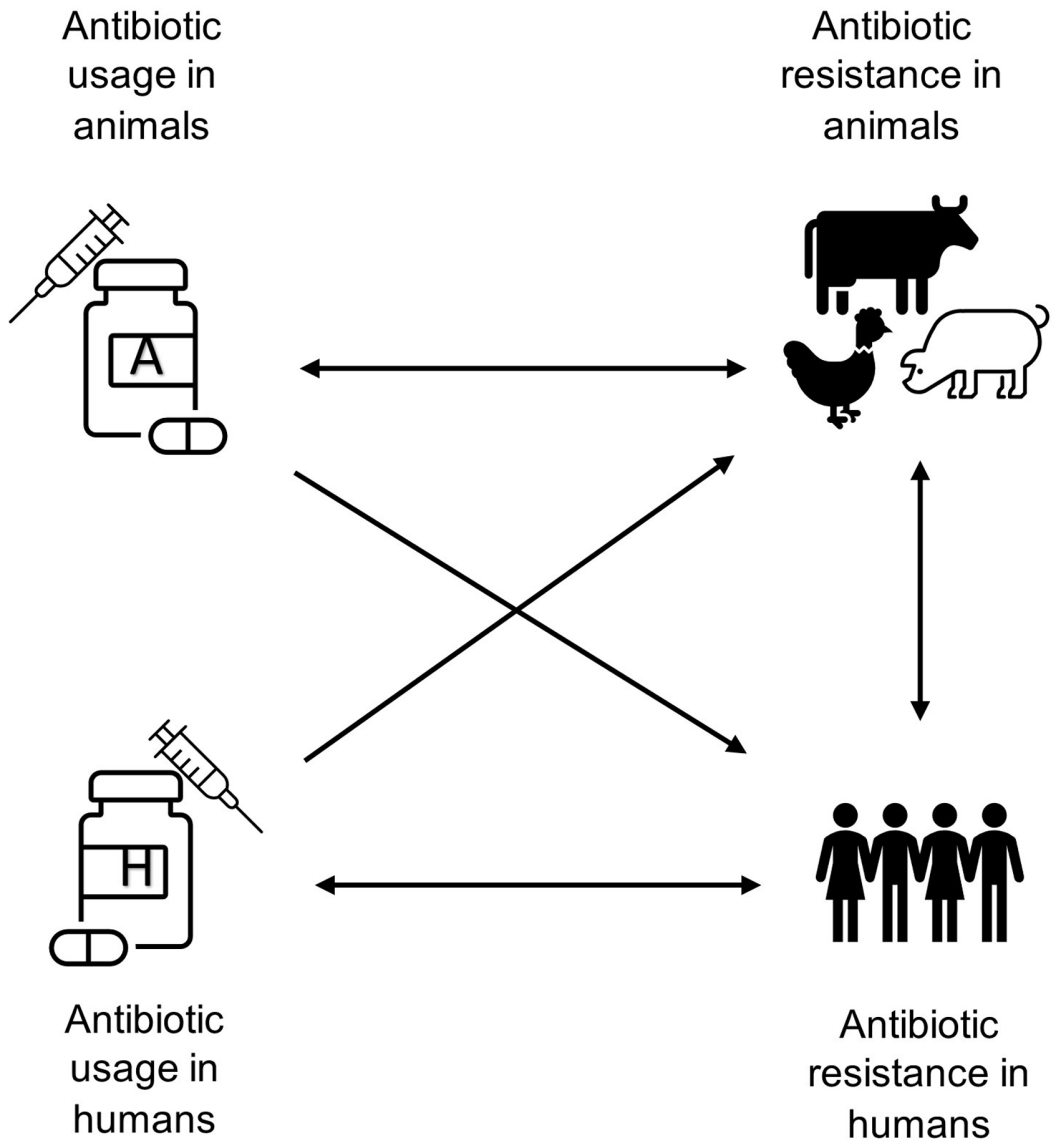
Schematic diagram highlighting the potential associations between antibiotic usage and antibiotic resistance in food-producing animals and humans.

This paper therefore undertakes an empirical analysis of the relationships between use of antibiotics in human and animal populations and antibiotic resistance in both populations using national surveillance data. We evaluate four possible effects: use in animals causing resistance in animals; use in animals causing resistance in humans; use in humans causing resistance in humans; and use in humans causing resistance in animals, as indicated in Figure 1. The existing evidence on these effects is extensive, but also incomplete. The most critical issues relate to cross-species effects on resistance. For example, bovine respiratory diseases lead to heavy consumption of antibiotics, which may lead to increased prevalence of resistant infections in both livestock and humans (13). While detailed studies show clearly that farmers and their direct contacts working with livestock acquire antibiotic resistance genes that seem clearly related to the use of antibiotics in these animals, what is less obvious is whether there is a wider spread in the human population (6). Our study provides evidence on this relationship. Moreover, there exists mixed evidence about the sharing of resistance genes across humans, livestock, and the environment (14, 15). Therefore, direct sharing of resistant infections is not the only way that usage of antibiotics in one population can affect others.

### 1.1. Animal–animal

The extensive use of antibiotics in livestock contributes to the emergence of antibiotic-resistant bacteria in animal populations (2, 16, 17). Numerous studies have investigated the link between antibiotic usage in food-producing animals and antibiotic resistance in bacteria present in those animals. Studies primarily focus on national surveillance reports from European countries for a variety of combinations of pathogens, antimicrobial substances, and animal species (2, 16–18). The evidence from these studies suggests that there is a positive correlation between the amount of antibiotics used and the development of resistance in bacteria present in food-producing animals. Studies also show that reducing antibiotic usage in food-producing animals could lead to reductions in resistance in those animals (19–23).

### 1.2. Animal–human

While it is believed that the widespread use of antibiotics in food-producing animals is a significant source of antibiotic resistance in humans, the specific impact on human health is poorly understood (6, 24, 25). Resistant bacteria could be transmitted to humans through the consumption of undercooked or raw food, cross-contamination with other foods, or indirectly through the environment (26). Ceftiofur use in chickens was tied to resistant infections in humans in the province of Quebec, Canada, suggesting that transmission occurred through handling of raw meat (27). Direct transmission from animals to farm workers is also a concern (28–30). Alternatively, antibiotics intended for animals may be excreted and their presence in the environment may increase human exposure to resistant bacteria (26, 31, 32). The relationship between antibiotic usage in animals and antibiotic resistance in humans has been studied in the literature mainly by focusing on transmission pathways and using molecular analysis (14, 15, 24). There exists evidence of transmission pathways of resistant bacteria from animals to humans, but the quantitative and ecological extent of the problem is not yet fully understood (33). Few studies have examined the direct impact of antibiotic use in animals on the occurrence of resistant bacteria in humans. The extent of the effect on humans outside of the farm is still poorly quantified (25).

### 1.3. Human–human

There have also been numerous studies of the effect of antibiotic use in humans on resistance in humans. Several studies have focused on specific populations, such as a nursing home or hospital, and demonstrated that increased antibiotic use tends to precede increases in resistance locally (34–37). At an ecological level, the studies are mostly cross-sectional, and therefore offer limited opportunities for inferring causality (7, 11, 38–41). Our recent research uses longitudinal data to show that increases in antibiotic use nationally are followed by persistent increases in resistance for at least 4 years (42).

### 1.4. Human–animal

To our knowledge, there is only one other study that examines the relationship between use of antibiotics by humans and resistance in animals (43). However, other evidence has suggested that resistance in the environment may be affected by human medicines. For example, recent evidence suggests that use of oseltamivir in humans may result in environmental exposure for birds that in turn develop oseltamivir-resistant avian influenza virus (44).

An important omission in these studies is the recognition that antibiotic use in humans and animals is occurring simultaneously. In this situation, it is reasonable to consider usage of antibiotics in both animals and humans can potentially affect resistance levels in all species at the same time. Use in animals, for example, may affect humans *directly* through excretion of antibiotics into the environment; but it may also affect humans *indirectly* through the increase in antibiotic resistance in animals who then interact with humans, sharing antibiotic resistance genes. And the resistance in humans could easily be transferred back to animals. Given these parallel mechanisms allowing resistance to spread, a true “One Health” approach requires a holistic approach which accounts for use in humans and in other animals.

This paper thus brings together comprehensive data on usage in humans and food-producing animals of 11 antibiotic classes and occurrence of resistance in three bacterial species common in humans and food-producing animals from European surveillance reports over 11 years in 31 countries. This allows us to make numerous contributions to the growing literature on antibiotic use and resistance.

First, although antibiotic usage occurs simultaneously in humans and other animals, existing studies have almost exclusively considered the effect of antibiotic use in humans *or* other animals. Our study introduces an analysis with usage in both humans and food-producing animals, allowing us to identify the marginal effect of usage in humans and animals separately. We are also able to estimate their joint effect. Adda attempted to do a similar analysis using data from US states (38). However, his study relied heavily on extrapolation and interpolation of data and lacked state-level data on antibiotic use in animals as well as resistance data from animal sources.^1^ Allel et al. conducted a recent cross-sectional study that considers usage in both humans and food-producing animals (43). While their analysis includes many more countries than ours, their data is cross-sectional in nature only and therefore cannot be used to address causality. We thus see these analyses as highly complementary and mutually reinforcing.

Second, our longitudinal data allows us to estimate the effect of usage on resistance in a causal framework, rather than just estimating correlations. Correlations can be informative, but it is hard to know from a cross-sectional analysis whether higher usage causes higher resistance or higher resistance causes higher usage. We use a methodology pioneered in the economics literature that allows us to bound the causal effect from use to resistance, though not to estimate it precisely.

Third, we show the effect of antibiotic usage in humans on resistance in food-producing animals at an ecological level. To our knowledge, only one other previous study has attempted to show how antibiotic use in humans is related to resistance in other animals (43).

## 2. Data and methods

### 2.1. Data on antibiotic resistance

Resistance data for our analysis is drawn mainly from the European Union Summary Report, published by the European Food and Safety Authority (EFSA), on antimicrobial resistance in zoonotic and indicator bacteria from humans, animals, and food, which is published annually. EFSA along with the European Centre for Disease Prevention and Control (ECDC) prepares the report on the occurrence of antimicrobial resistance in isolates from human cases and in isolates from animals and foodstuffs. Participating laboratories of both member and non-member states of the European Union (EU) report their data on antimicrobial resistance. ECDC’s protocols for harmonized monitoring and reporting of resistance in humans and food-producing animals are followed, during the reporting process, to overcome challenges of comparing antimicrobial resistance data from various countries using different laboratory methods and different criteria for interpreting resistance (45).

The reports provide resistance data for humans to specific antibiotic molecules for two important zoonotic pathogens, *Salmonella*, and *Campylobacter*. For *Salmonella*, data on total number of isolates tested and number of resistant isolates are available for several selected serovars of importance. The reports also provide similar data for two most important *Campylobacter* species. These reports, however, did not include data on resistance to *Escherichia coli* in humans. For this bacterial species, we therefore used data from the ECDC Surveillance Atlas of Infectious Diseases (46). We aggregated these total number of tested isolates and number of resistant isolates from different species of these pathogens, that were tested against specific antibiotics, at the genus level for each year and country. Then, using the total number of tested isolates and number of resistant isolates for each bacteria-antibiotic combination, we calculated the percentage of resistant isolates for each year and country. Data on human isolates were not present in the annual reports for the years 2008 and 2018. Thus, our data on antibiotic resistance in humans, given by the percentage of resistant isolates, vary by country, bacteria, and antibiotic class over the years 2009–2017.

On the animal side, resistance data to specific antibiotics molecules for *Salmonella*, *Campylobacter* and *Escherichia coli* were used. These are present in the EFSA annual summary reports (45). For *Salmonella and Campylobacter* resistance data is available for selected important species. Moreover, the isolates from different species of these pathogens originated from multiple sources, including live fowl, cattle, pigs and meat from the same species. The data across all these different sources from different bacterial species were aggregated at the genus level for each year and country. The data for animals is available for the years 2008–2018, but during this reporting period the sampling from the sources is not consistent across years. We calculated the percentage of resistant isolates using the total number of isolates and number of resistant isolates for 3 bacteria genera tested against specific antibiotics, for each year and country. Data on antibiotic resistance for food-producing animals vary by country, bacteria, and antibiotic combination and is available from 2008 to 2018.

Epidemiological cut-off values and clinical breakpoints are used to interpret resistance in human isolates from minimum inhibitory concentration (MIC) data. MIC refers to the lowest concentration of an antimicrobial agent that is required to inhibit the growth of a microorganism under standard laboratory conditions (47). This measure is used to determine the susceptibility of microorganisms to antibiotics applying different methods, such as disk diffusion and broth dilution. A particular specimen is defined as resistant if it crosses a certain threshold of the MIC ratio. The annual reports use thresholds defined by the European Committee on Antimicrobial Susceptibility Testing (EUCAST) guidelines for detection of resistance for bacteria-antibiotic combinations included in this study (48, 49).

### 2.2. Data on antibiotic usage

Antibiotic usage data for humans were extracted from the IQVIA MIDAS database. IQVIA reports the total volume of sales of antibiotic molecules used in human medicine based on national surveys. We included only the antibiotic molecules that were also present in resistance data. Antibiotic molecules were then aggregated by class (e.g., fluoroquinolones) and quantified in metric tonnes. Thus, human antibiotic usage in tonnes varies by country and class and is available annually for the period 2008–2018. We extracted data on antibiotic usage in food-producing animals from the ESVAC database. The ESVAC project collects sales data of veterinary antimicrobials in participating European countries. The data is in tonnes for antibiotic class for 27 member states of the EU and 4 non-member states from 2008 to 2018 (50). Usage data for animals is available for 10 antibiotic classes, and, for humans for the same 10 classes plus *Carbapenems*.

### 2.3. Complementary data

We complement our data with control variables that vary by year and country. The control variables included are Population Correction Units for animals drawn from the ESVAC database (50), human population, Gross Domestic Product, and health expenditure *per capita* drawn from the World Bank Databank (51, 52), and the Corruption Perception Index from Transparency International (53). The Population Correction Unit is a standardized average weight in kilograms of all animals at the time of antibiotic treatment multiplied by the number of animals based on national statistics (50).

### 2.4. Sample definition

Merging these sets of data gives us an 11-year panel of data on usage in 11 antibiotic classes and on measured resistance to each class in 3 bacterial species in humans and food-producing animals in 31 European countries. In the early years, not all countries report, and over time the number of reporting countries increases. In addition, not all countries report resistance data on every combination of bacteria and antibiotic class for each year. Moreover, usage data is also not available for every member and non-member states for all years. This makes it an unbalanced panel. It should be noted that resistance data varies by year, country, bacteria, and antibiotic class, whereas usage data varies only by year, country, and class.

## 3. Methodology

### 3.1. Summary statistics and plots

We begin by summarizing the raw data. We calculate total usage in tonnes in humans and animals by class of antibiotics and find the average across years; and we calculate average resistance as the percentage of samples meeting ESVAC’s resistance threshold by bacterial species.

We then show this data with greater granularity by plotting the relationship between log transformed resistance and usage data, using binned scatter plots and lines of best fit. To generate a binned scatterplot, the x-axis variable is grouped into equal-sized bins, then the means of the variables along both horizontal and vertical dimensions are computed within each bin. These means are used to create a scatter plot. We use the “binsreg” package in R to automatically determine bin sizes and compute corresponding means. Lines of best fit are plotted using ordinary least squares method, which provides the best linear approximation to the conditional expectation function. The plots explore the relationship between resistance and usage for food-producing animals and humans separately. First, we pool the data for an overview of the relationships and then explore relations by year, bacteria and class. These associations are also illustrated for each country in Supplementary material. The literature has extensive evidence on such correlation measures. However, the evidence is predominantly drawn from cross-sectional studies without accounting for time and controlling for confounding factors. Moreover, the findings are only relevant and limited to the particular samples and environments studied (2, 18, 29).

### 3.2. Regression analysis

Our main dependent variable is the natural log of the prevalence of resistance, as given by the percentage of resistant isolates. We estimate models separately using resistance data for food-producing animals and humans. The natural log of antimicrobial usage in tonnes, in food-producing animals and humans are our two main explanatory variables for identifying the marginal resistance effects arising from simultaneous usage. In addition, we also use the sum of antibiotic usage in food-producing animals and humans as the main explanatory variable in an alternative model which estimates the effect of combined usage on resistance for animals and humans.

To isolate the causal effect of antibiotic usage on antibiotic resistance we employ fixed effects and lagged dependent variable models. The effects on resistance from food-producing animals and humans are isolated separately. The models are presented below:

Fixed-effect specification:


$$\begin{array}{l} \ln\left({\text{Resistance}}_{i,t,g}\right)=\alpha +\beta \;\ln\left(\text{Animal}\_\text{Us}e_{i,t}\right)\\ \;\;\;\;\;\;\;\;\;\;\;\;\;\;\;\;\;\;\;\;\;\;\;\;\;\;\;\;\;\;\;\;\;\;\;\;\;\;+\gamma \;\ln\left(\text{Human}\_\text{Us}e_{i,t}\right)+\theta_{i}\\ \;\;\;\;\;\;\;\;\;\;\;\;\;\;\;\;\;\;\;\;\;\;\;\;\;\;\;\;\;\;\;\;\;\;\;\;\;\;+\lambda_{t}+X_{i,t}\;\;+\varepsilon_{i,t} \end{array}$$

Lagged-dependent variable (LDV) specification:


$$\begin{array}{l} \ln\left({\text{Resistance}}_{i,t,g}\right)=\alpha +\beta \;\ln\left(\text{Animal}\_\text{Us}e_{i,t}\right)+\gamma \;\ln\left(\text{Human}\_\text{Us}e_{i,t}\right)\\ \;\;\;\;\;\;\;\;\;\;\;\;\;\;\;\;\;\;\;\;\;\;\;\;\;\;\;\;\;\;\;\;\;\;\;\;\;\;\;\;\;+\delta \ln\left({\text{Resistance}}_{i,t-1}\right)+\lambda_{t}+X_{i,t}+\varepsilon_{i,t} \end{array}$$

where ${\text{Resistance}}_{i,t,g}$ is the prevalence of resistance for unit for unit i, in year t, for “groups” g indicating either humans or food-producing animals. A unit i is formed by means of stratifying our data by country, bacteria, and antibiotic. The explanatory variables of interest in both the models are usage in food-producing animals and humans for unit i, in year t, denoted by $\text{Animal}\_\text{Us}e_{i,t}$ and $\text{Human}\_\text{Us}e_{i,t}$ respectively.

We include unit fixed effects in FE estimation, denoted by $\theta_{i}$, to account for unobserved characteristics that are specific to units or different stratifications and are constant over time. Both estimation strategies include year ($\lambda_{t})$ fixed effects, which controls for unobserved variables that are specific to a particular year but shared across countries. In LDV estimation, instead of using unit fixed effects $\left(\theta_{i}\right)$ we use one-year lagged dependent variable denoted by ${\text{Resistance}}_{i,t-1}$. This strategy accounts for the fact that unobserved unit or group characteristics may not be fixed over time and, instead, past resistance values influence the current value of resistance. In other words, this model is designed to model past resistance as a time-varying confounder which cannot be controlled for by using fixed effects. $X_{i,t}$ is a vector of country-and year-specific controls: population Correction Unit (PCU) and total human population. PCU for food-producing animals and total population of humans take into account difference in sizes and structure of the food-producing animal population and human population in each European country. We add Gross Domestic Product, health expenditure *per capita*, and the Corruption Perception Index as additional covariates in the sensitivity analysis section, but we do not anticipate these control variables to have a substantial effect on the coefficients of interest, given the use of country fixed effects or the lagged dependent variable. The error term is given by $\varepsilon_{i,t}$. Coefficient β measures the effect of antibiotic usage in food-producing animals on resistance. Similarly, coefficient γ measures the effect of antibiotic usage in humans on resistance. Both coefficients should be interpreted as elasticities, i.e., the percentage increase (decrease) in resistance correlated with a percentage increase (decrease) in usage. The models using resistance in humans and resistance in food-producing animals are estimated separately.

The use of the two specifications enables us to check the robustness of our findings using alternative identifying assumptions. That is, findings from both specifications should be broadly similar. Moreover, according to Angrist and Pischke (54), fixed effects and lagged dependent variable estimates have a useful bracketing property. As they show, the LDV specification provides the lower bracket while the FE specification provides the upper bracket. Thus, using these two specifications enables us to bound the causal effect.

Furthermore, we carry out sensitivity analysis of our findings based on these two specifications. First, to test for robustness of these results, we replicate these regressions after lagging the usage variables by 1 or 2 years. Second, we alter the sample definition, excluding outliers and including only specific bacteria. Third, we include covariates that have been shown to be related to antibiotic usage or resistance (7, 38, 55). Fourth, we ran these regressions after excluding small countries (those with a population under 6 million people) as a further robustness test. If our estimation strategy is sound, we anticipate that the results using different samples and additional covariates should not differ much from our main results.

## 4. Results

Table 1 presents average antibiotic usage, given in tonnes, by class. *Tetracyclines* are the most heavily used antibiotics class in food-producing animals while in humans, *Penicillins* are most used. Our data does not include *Carbapenem* use in animals. Given the high variation in use across different antibiotics, we tested exclusion of heavily and lightly used antibiotics, as described below.

**Table 1:** Antibiotic usage by class for food-producing animals and humans.

Class	Average animal usage in tonnes	Avg. human usage in tonnes
Aminoglycosides	12.46	0.84
Amphenicols	3.68	0.99
Carbapenems	-	3.86
Cephalosporins	5.77	3.56
Fluoroquinolones	8.97	81.65
Macrolides	21.70	138.90
Penicillins	73.04	2237.31
Polymyxins	16.19	20.00
Sulfonamides	32.34	109.07
Tetracyclines	107.38	19.11
Trimethoprim	4.77	37.77

As seen in Table 2, *Campylobacter* exhibits the highest average resistance in food-producing animals followed by *Escherichia coli* and *Salmonella*. In humans, *Campylobacter* also exhibits the highest resistance followed by *Escherichia coli*. The distribution plots and central tendency tables in Supplementary Tables S1, S2; Supplementary Figures S1–S4 show that the data on resistance and usage vary widely. Since we anticipate that the relationship between usage and resistance is most likely to be related to percentage changes, rather than unit changes, we log transform these variables. This also makes the range of the variables much more compact.

**Table 2:** Antibiotic resistance by bacteria in food-producing animals and humans.

Bacteria	Average resistance (%) in animals	Average resistance (%) in humans
Campylobacter	24.64	27.22
Escherichia	18.31	25.12
Salmonella	12.85	13.36

**Figure 2:**
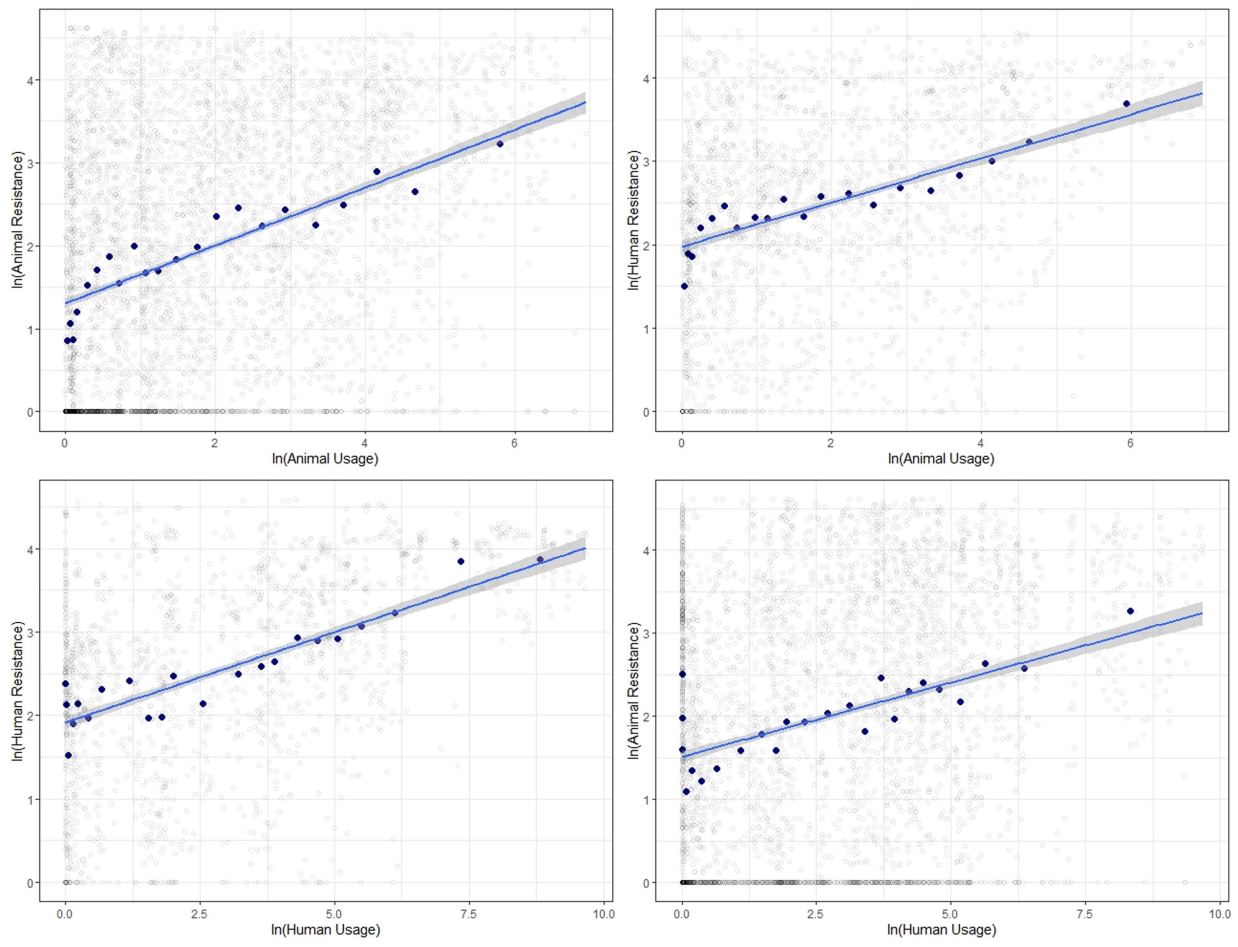
Binned scatter plots along with lines of best fit between animal usage and animal resistance (top-left), animal usage and human resistance (top-right), human usage and human resistance (bottom-left) and human usage and animal resistance (bottom-right). The light gray circles show the raw data.

The correlations between log-transformed antibiotic usage and resistance measures for food-producing animals and humans generally show a positive relationship. Figure 2 presents the scatter plots along with the line of best fit showing the 95% confidence interval with all data pooled. We find strong positive correlations between usage and resistance for both animals and humans and between the two.

**Figure 3:**
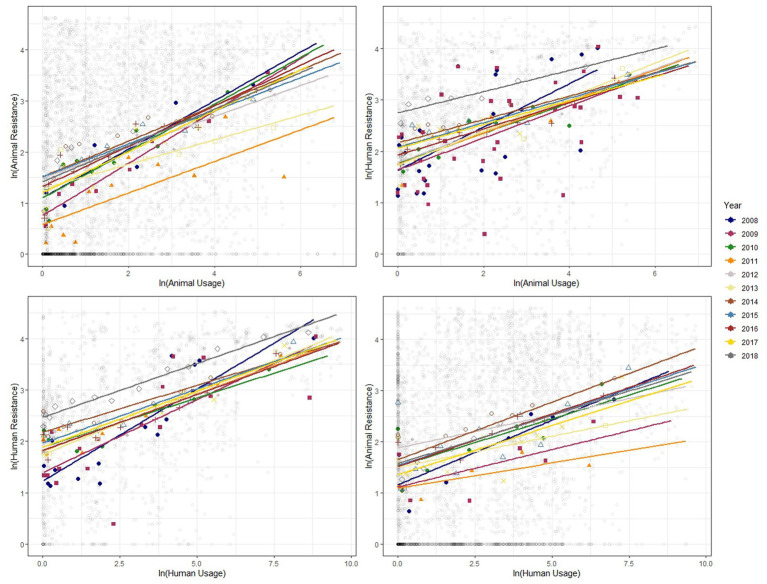
Binned scatter plots along with lines of best fit for years 2008–2018, between animal usage and animal resistance (top-left), animal usage and human resistance (top-right), human usage and human resistance (bottom-left) and human usage and animal resistance (bottom-right). The light gray circles show the raw data.

**Figure 4:**
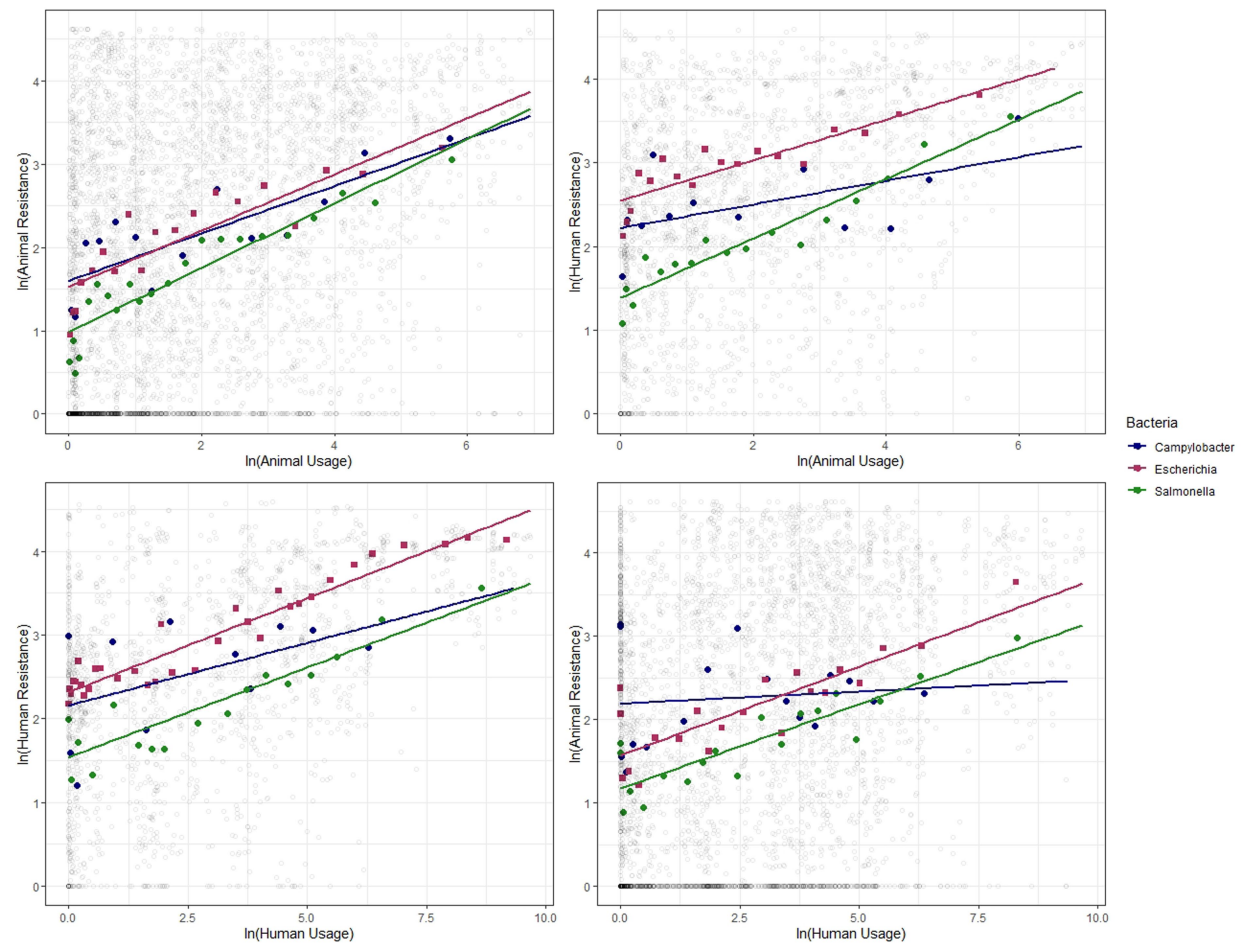
Binned scatter plots along with lines of best fit for the 3 bacteria genera, between animal usage and animal resistance (top-left), animal usage and human resistance (top-right), human usage and human resistance (bottom-left) and human usage and animal resistance (bottom-right). The light gray circles show the raw data.

**Figure 5:**
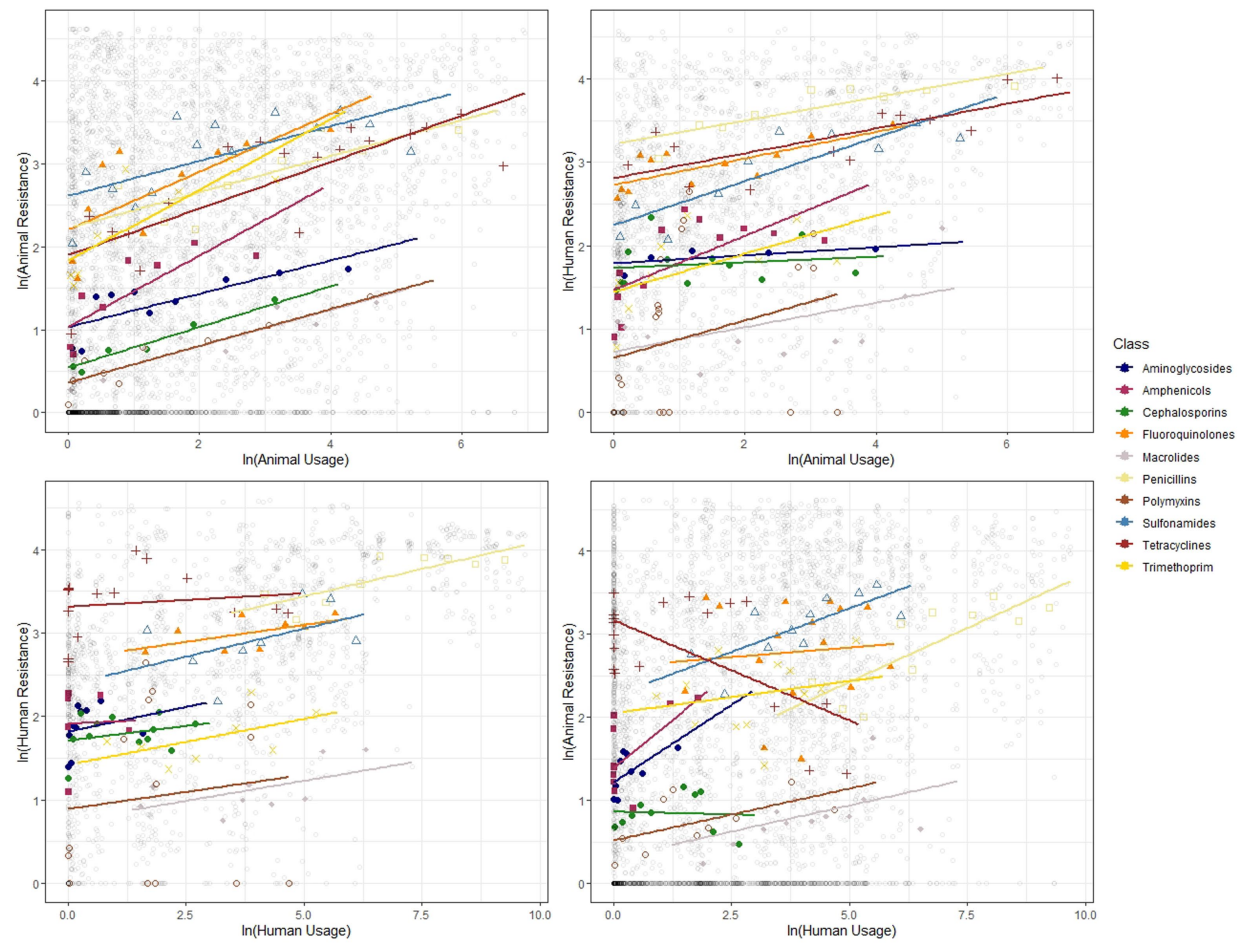
Binned scatter plots along with lines of best fit for the 11 antibiotic classes, between animal usage and animal resistance (top-left), animal usage and human resistance (top-right), human usage and human resistance (bottom-left) and human usage and animal resistance (bottom-right). The light gray circles show the raw data.

We show similar plots with the data disaggregated by year (Figure 3), bacteria (Figure 4) and antibiotic class (Figure 5). The strength of these associations varies but we find positive correlations between usage and resistance across all years, bacteria and classes (except for a negative correlation between human usage and animal resistance for the two antibiotic classes, *Cephalosporins* and *Tetracyclines*). We go a step further and disaggregate these relationships by year and by bacteria for each country. Results are presented in Supplementary Figures S5, S6. Associations between resistance from animals and humans also exhibit positive correlations. The same is true for associations between usage from the two (Supplementary Figure S7).

These plots reveal positive correlations between antibiotic usage and the prevalence of resistance for a given year, country, bacteria and antibiotic class. The positive association exists within the same group and across groups. That is, usage of antibiotics in animals is positively related to occurrence of resistance in animals and in humans. Similarly, human usage of antibiotics is positively related to resistance levels in humans and in animals.

After exploring the correlations described above, we estimate the relationship between antibiotic usage and resistance using regression analysis. The results from our models are presented in Table 3. Columns 1 and 2 show estimated elasticities relating to resistance in animals, and columns 3 and 4 show those for resistance in humans. As described above, these relationships can be interpreted as indicating the lower and upper bounds of a causal effect. Columns 1 and 3 form the lower bounds for resistance effects and columns 2 and 4 form the upper bounds. We find that a 1% increase in antibiotic usage in animals increases resistance in animals between 0.22% and 0.41% and in humans between 0.03% and 0.40%. In addition, a 1% increase in antibiotic usage in humans leads to an increase in resistance in animals between 0.06% and 0.13% and in humans between 0.03% and 0.16%. All the coefficients, whether lower or upper bounds, are statistically significant. It is evident from these findings that an increase in antibiotic usage in both animals and humans contributes to an increase in resistance in animals and people.

**Table 3:** Simultaneous usage effects of antibiotics on animal and human resistance.

	(1)	(2)	(3)	(4)
Variables	*ln* (animal resistance)		*ln* (human resistance)	
*ln* (animal usage)	0.224^***^ (0.0178)	0.408^***^ (0.0205)	0.0306^**^ (0.0117)	0.397^***^ (0.0230)
*ln* (human usage)	0.0647^***^ (0.00874)	0.133^***^ (0.00903)	0.0283^***^ (0.00600)	0.157^***^ (0.00896)
Constant	1.001 (0.577)	35.61 (23.99)	0.836^**^ (0.318)	73.02^**^ (28.02)
Observations	3,062	3,983	1,562	1818
R-squared	0.486	0.273	0.800	0.364
Year FE	YES	YES	YES	YES
Country FE	NO	YES	NO	YES
Lagged dependent variable	YES	NO	YES	NO

Standard errors in parentheses ^*^p < 0.05, ^**^p < 0.01, ^***^p < 0.001.

The cross-species effect of usage on resistance is particularly important in this analysis. It is clear that higher usage can lead to higher resistance, but causality can also flow in the other direction, since with higher resistance, more antibiotics might be used to treat an infection. However, there is no direct mechanism by which higher prevalence of resistance in animals should lead to more intensive use of antibiotics in people. The cross-species effects are therefore naturally interpreted as being causal.

Table 4 shows the results of regressions in which human and animal use of antibiotics is summed, rather than being treated separately. We find consistent results. Generally, the upper and lower bounds are somewhat tighter in these regressions.

**Table 4:** Combined usage effects of antibiotics on animal and human resistance.

	(1)	(2)	(3)	(4)
Variables	*ln* (animal resistance)		*ln* (human resistance)	
*ln* (combined usage)	0.139^***^ (0.0109)	0.274^***^ (0.00950)	0.0420^***^ (0.00782)	0.296^***^ (0.00887)
Constant	−0.374 (0.542)	43.64 (24.02)	0.800^**^ (0.292)	76.38^**^ (27.30)
Observations	3,062	3,983	1,562	1818
R-squared	0.479	0.271	0.800	0.367
Year FE	YES	YES	YES	YES
Country FE	NO	YES	NO	YES
Lagged dependent variable	YES	NO	YES	NO

Standard errors in parentheses ^*^p < 0.05, ^**^p < 0.01, ^***^p < 0.001.

To further investigate the causal effects, we go a step further. Instead of using contemporaneous usage we lag the usage variables by 1 and 2 periods. This addresses the problem of reverse causality, since resistance in the current year should not have any effect on usage in preceding years. The results are presented in Table 5. Columns 1–2 and 5–6 display the bounded effect on animal and human resistance respectively, from usages lagged by one year. We find strong and statistically significant evidence of usage driving resistance across and within humans and food-producing animals. The results are quite similar when the usage variables are lagged 2 years as shown in columns 3–4 and columns 7–8. Lagging the usage variables results in estimated coefficients that are broadly in line with the contemporaneous usage data.

**Table 5:** Simultaneous effects of lagged antibiotic usage on animal and human resistance.

	(1)	(2)	(3)	(4)	(5)	(6)	(7)	(8)
Variables	*ln* (animal resistance)				*ln* (human resistance)			
*ln* (animal usage)	0.188^***^ (0.0178)	0.414^***^ (0.0215)	0.280^***^ (0.0226)	0.425^***^ (0.0227)	0.0366^*^ (0.0161)	0.386^***^ (0.0256)	0.0569^**^ (0.0192)	0.399^***^ (0.0262)
*ln* (human usage)	0.0610^***^ (0.00853)	0.147^***^ (0.00961)	0.105^***^ (0.0111)	0.146^***^ (0.0102)	0.0417^***^ (0.00756)	0.160^***^ (0.00980)	0.0585^***^ (0.00868)	0.161^***^ (0.0102)
Constant	1.656^**^ (0.527)	31.82 (31.69)	1.190 (0.742)	−15.25 (39.59)	1.068^*^ (0.417)	90.18^**^ (33.26)	1.380^**^ (0.512)	117.1^**^ (44.11)
Observations	2,325	3,046	1,828	2,568	1,277	1,572	1,012	1,321
R-squared	0.615	0.310	0.446	0.298	0.745	0.358	0.734	0.377
Year FE	YES	YES	YES	YES	YES	YES	YES	YES
Country FE	NO	YES	NO	YES	NO	YES	NO	YES
Lagged dependent variable	YES	NO	YES	NO	YES	NO	YES	NO
Usage variables lagged 1 year	YES	YES	NO	NO	YES	YES	NO	NO
Usage variables lagged 2 years	NO	NO	YES	YES	NO	NO	YES	YES

Standard errors in parentheses ^*^p < 0.05, ^**^p < 0.01, ^***^p < 0.001.

### 4.1. Further robustness checks

To check for robustness of our results we estimate both models after excluding possible outliers. We exclude penicillin, aminoglycosides and amphenicols separately from our data and run the models. As shown in Table 1, penicillin is the most used antibiotic class in humans and aminoglycosides and amphenicols are the least used classes. Therefore, we treat them as outliers and exclude them separately from our data. In addition, we also run the models separately for individual bacteria, by including additional control variables and by excluding countries with a population less than 6 million people. The results for this sensitivity analysis are presented in Supplementary Tables S3–S6.

Sensitivity analysis reveals our results are robust to different sample definitions. When the sample size is restricted by including only one bacterial species at a time, the statistical significance is naturally lower. The effects are always statistically significant for the fixed effect estimates, but not necessarily for the LDV specification. The range of resistance effects when we exclude countries with populations less than 6 million people are also robust and close to estimates in Tables 3, 4.

## 5. Discussion

The inherent convolution of growing antimicrobial resistance makes it difficult to understand the exact ways in which it spreads among domesticated animals, humans, and the environment. This paper aims to shed light on the relationships between usage and resistance between and across human and food-producing animal populations in Europe. It highlights relationships that have not been covered extensively in the existing literature, specifically, the effect of usage in animals on human resistance and the effect of usage in humans on animal resistance. Using data from European surveillance reports, the paper shows that simultaneous and combined antibiotic usage in food-producing animals and humans have a positive impact on the incidence of resistance in both the populations.

What is novel in our analysis is that we simultaneously include both human and animal use of antibiotics when examining the relationships with resistance. This enables us to observe the marginal relationship between antibiotic use and resistance in humans and animals separately, conditional on use in the other group. Moreover, our econometric approach enables us to infer causality at an ecological level. That greater use of antibiotics would increase the prevalence of resistance is of course not surprising: however, we are able to quantify this effect within a range. In addition, we are able to show for the first time the effect of human use of antibiotics on the prevalence of resistance in food-producing animals.

The estimated effects are both substantial and statistically significant. Strikingly, the lower and upper bounds of the effect of antibiotic use in animals on resistance in humans are not smaller than the effect of antibiotic use in humans. The estimated elasticities are, from the perspective of long-term impact on resistance, disturbingly large. Even at the lower bound, an increase in antibiotic use in animals of only 10% is expected to increase the prevalence of resistance in animals by around 2% and in humans by around 0.3%. Since, as we show in a recent paper, resistance tends to persist over a period of years, increases in usage may lead to long-term increases in resistance (42).

Our study has numerous limitations. We are only able to provide the average effects of usage on resistance for Europe as a nation. Our data do not allow us to explicate clear mechanisms of how usage affects resistance within or across human and food-producing animal populations. There are several factors at play that determine the occurrence of resistance against antibiotics among bacteria. This includes the ancient molecular mechanisms behind the emergence of resistant bacteria and the natural concentration of antibiotics and resistant genes in the environment (56–58). It would be ideal to consider all the factors that contribute to the evolution of antibiotic resistance in bacteria; however, appropriate measures for the impact of environmental factors are limited and there is a disconnect in surveillance data for humans and animals (59, 60).

Antibiotic usage has been linked to an increase in resistance in both humans and food-producing animals, making it a critical public health issue. Injudicious use and over-use of antibiotics within and across clinical and agricultural settings provides a favorable environment for the emergence of antibiotic resistant bacteria, causing infections that are difficult to treat (61). The potential health implications of these are significant, as antibiotic resistant bacteria can spread from food-producing animals to humans and *vice-versa*, resulting in increased morbidity and mortality (62, 63). In light of this matter, policymakers have a crucial role to play in addressing antibiotic resistance. A recent study projects the use of antibiotics in animal farming to increase by 8% between 2020 and 2030 (64). One immediate measure is to implement policies aimed at curbing and promoting judicious use of antibiotics in human medicine and animal production. However, as we show in a previous study, decreasing antibiotics use alone may not be a sufficient solution (42). Along with judicious antibiotic use, development of alternative technologies, including using innovative financial mechanisms such as the UK’s antibiotic subscription pilot may be necessary (65, 66). Moreover, policies to encourage farmers and healthcare providers to adopt preventive measures, such as improved hygiene and vaccination, could potentially reduce the need for antibiotics and mitigate the development of resistance (67). Therefore, addressing this issue requires a multi-disciplinary approach that involves stakeholders from all relevant sectors and recognizing the health of animals, humans and the environment are interwoven.

It is notable that this study’s data are drawn from European countries, which tend to have relatively low rates of antibiotic usage and resistance (68). Since antibiotic resistance is a more pressing problem in many low-and middle-income countries, it would be useful to better understand the relationship between usage and resistance in those countries (69, 70). Understanding the effect of antibiotic consumption on rates of resistance is of great importance and will require ongoing investment into consistent surveillance data on a global scale.

## 6. Conclusion

To summarize, this paper provides new insights into the complex relationships between antibiotic usage and resistance in humans and food-producing animal populations in Europe. Our analysis reveals that usage of antibiotics by both humans and food-producing animals has a significant and statistically relevant effect on the rates of resistance in both groups. The estimated own-and cross-elasticities are worrying and highlight the potential long-term impacts of antibiotic usage on resistance. However, the study has limitations, including the lack of clear mechanisms explaining the relationship between usage and resistance and the inability to account for environmental factors. Antibiotic resistance is a critical public health concern, and policymakers need to promptly adapt a multi-disciplinary approach which engages all relevant stakeholders and acknowledges the interdependence of animal, human and environmental health. Simultaneous usage of antibiotics in various sectors and direct and indirect sharing of resistance across humans, animals and environment calls for a need to implement integrated strategies to monitor usage and resistance across heterogenous One Health dominions.

## Data availability statement

Publicly available datasets were analyzed in this study. This data can be found at: PRISM Data: University of Calgary’s Data Repository—Replication Data for: the effect of antibiotic usage on resistance in humans and food-producing animals: a longitudinal, One Health analysis using European data, doi: 10.5683/SP3/RXHWFP.

## Author contributions

SR and AH: conceptualization, data verification, investigation, methodology, writing, and review and edit. SR: literature review, data curation, and formal analysis. AH: funding acquisition and supervision. All authors contributed to the article and approved the submitted version.

## Funding

AH’s work was funded by Canadian Institute for Health Research [Grant #170388], Canadian Institute for Health Research [Grant #387609] through the Global 1 Health Network, and Major Innovation Fund program of the Ministry of Jobs, Economy and Innovation, Government of Alberta through the AMR—One Health Consortium.

## Conflict of interest

The authors declare that the research was conducted in the absence of any commercial or financial relationships that could be construed as a potential conflict of interest.

## Publisher’s note

All claims expressed in this article are solely those of the authors and do not necessarily represent those of their affiliated organizations, or those of the publisher, the editors and the reviewers. Any product that may be evaluated in this article, or claim that may be made by its manufacturer, is not guaranteed or endorsed by the publisher.

## Footnotes

^1^To be more precise, Adda lacked state-level data on usage of antibiotics in animals and therefore projected national data onto each state using the value of sales of different food-producing animals. He did not attempt to assess the impact of antibiotic usage on resistance in animals.
