# Supplementary material for: The effect of antibiotic usage on resistance in humans and food-producing animals: a longitudinal, One Health analysis using European data
Source: Front Public Health. 2023 Jun 15;11:1170426. doi: 10.3389/fpubh.2023.1170426 (PMC10311110; doi:10.3389/fpubh.2023.1170426)
Supplement: Supplementary file 1 [file Data_Sheet_1.docx]

Supplementary Material

The effect of antibiotic usage on resistance in humans and food-producing animals: a longitudinal, One Health analysis using European data

Sakib Rahman^1^, Aidan Hollis^1*^

*** Correspondence:** Aidan Hollis: ahollis@ucalgary.ca

# Supplementary Figures and Tables

For more information on Supplementary Material and for details on the different file types accepted, please see [here](https://www.frontiersin.org/guidelines/author-guidelines#supplementary-material).

## Supplementary Tables

| Table S1: Summary Statistics of Antibiotic Usage by Class for Food-Producing Animals and Humans | | | | | | | | | | |
| --- | --- | --- | --- | --- | --- | --- | --- | --- | --- | --- |
| Class | **Animal Usage in Tonnes** | | | | | **Human Usage in Tonnes** | | | | |
|  | **Minimum** | **Average** | **Median** | **Std. Deviation** | **Maximum** | **Minimum** | **Average** | **Median** | **Std. Deviation** | **Maximum** |
| Aminoglycosides | 0.01 | 12.46 | 2.01 | 27.37 | 204.97 | 0.00 | 0.84 | 0.22 | 2.57 | 22.18 |
| Amphenicols | 0.00 | 3.68 | 1.03 | 6.72 | 47.86 | 0.00 | 0.99 | 0.00 | 2.00 | 9.67 |
| Carbapenems | - | - | - | - |  | 0.00 | 3.86 | 2.31 | 5.81 | 35.00 |
| Cephalosporins | 0.00 | 5.77 | 1.09 | 10.73 | 60.05 | 0.00 | 3.56 | 1.21 | 4.54 | 33.53 |
| Fluoroquinolones | 0.01 | 8.97 | 1.75 | 18.71 | 99.61 | 1.89 | 81.65 | 47.20 | 90.63 | 383.53 |
| Macrolides | 0.00 | 21.70 | 4.65 | 36.73 | 180.71 | 2.43 | 138.90 | 44.49 | 261.08 | 2053.13 |
| Penicillins | 0.11 | 73.04 | 15.35 | 138.76 | 697.72 | 29.90 | 2237.31 | 652.72 | 3223.18 | 15912.65 |
| Polymyxins | 0.00 | 16.19 | 1.10 | 42.25 | 263.00 | 0.00 | 20.00 | 2.47 | 52.34 | 532.51 |
| Sulfonamides | 0.01 | 32.34 | 7.29 | 54.88 | 341.96 | 1.13 | 109.07 | 54.51 | 134.31 | 541.92 |
| Tetracyclines | 0.01 | 107.38 | 29.87 | 192.62 | 1043.12 | 0.00 | 19.11 | 0.97 | 36.94 | 178.97 |
| Trimethoprim | 0.01 | 4.77 | 0.97 | 8.95 | 66.65 | 0.23 | 37.77 | 18.04 | 55.14 | 298.51 |

| Table S2: Summary Statistics of Antibiotic Resistance by Bacteria for Food-Producing Animals and Humans | | | | | | | | | | |
| --- | --- | --- | --- | --- | --- | --- | --- | --- | --- | --- |
| Bacteria | **Animal Resistance** | | | | | **Human Resistance** | | | | |
|  | **Minimum** | **Average** | **Median** | **Std. Deviation** | **Maximum** | **Minimum** | **Average** | **Median** | **Std. Deviation** | **Maximum** |
| Campylobacter | 0.00 | 24.64 | 7.41 | 30.03 | 100.00 | 0.00 | 27.22 | 15.69 | 28.78 | 97.67 |
| Escherichia | 0.00 | 18.31 | 8.38 | 22.13 | 100.00 | 2.11 | 25.12 | 16.71 | 19.54 | 77.96 |
| Salmonella | 0.00 | 12.85 | 4.61 | 18.69 | 100.00 | 0.00 | 13.36 | 7.01 | 16.35 | 95.70 |

| Table S3: Simultaneous effects of lagged antibiotic usage on animal and human resistance excluding certain antibiotic classes. | | | | |
| --- | --- | --- | --- | --- |
| Excluding Penicillin | | | | |
|  | (1) | (2) | (3) | (4) |
| Variables | ln (Animal Resistance) | | ln (Human Resistance) | |
| ln (Animal Usage) | 0.185***  (0.0195) | 0.444***  (0.0230) | 0.0375  (0.0198) | 0.387***  (0.0303) |
| ln (Human Usage) | 0.0623***  (0.0118) | 0.171***  (0.0143) | 0.0395**  (0.0133) | 0.159***  (0.0168) |
| Constant | 1.704**  (0.581) | 26.61  (34.60) | 0.981  (0.502) | 77.66*  (37.46) |
| Observations | 2078 | 2728 | 1061 | 1314 |
| R-squared | 0.603 | 0.271 | 0.690 | 0.224 |
| Excluding Aminoglycosides | | | | |
| ln (Animal Usage) | 0.168***  (0.0185) | 0.417***  (0.0220) | 0.0449**  (0.0171) | 0.411***  (0.0250) |
| ln (Human Usage) | 0.0475***  (0.00912) | 0.140***  (0.0105) | 0.0214**  (0.00780) | 0.130***  (0.0106) |
| Constant | 1.221*  (0.553) | 39.26  (34.84) | 1.350**  (0.446) | 86.07*  (38.02) |
| Observations | 2013 | 2638 | 1041 | 1279 |
| R-squared | 0.643 | 0.306 | 0.745 | 0.336 |
| Excluding Amphenicols | | | | |
| ln (Animal Usage) | 0.190***  (0.0181) | 0.431***  (0.0220) | 0.0378*  (0.0162) | 0.401***  (0.0259) |
| ln (Human Usage) | 0.0643***  (0.00872) | 0.156***  (0.00967) | 0.0422***  (0.00760) | 0.164***  (0.00983) |
| Constant | 1.829***  (0.545) | 34.75  (32.28) | 1.062*  (0.420) | 89.03**  (33.32) |
| Observations | 2227 | 2920 | 1245 | 1534 |
| R-squared | 0.618 | 0.317 | 0.756 | 0.363 |
| Year FE | YES | YES | YES | YES |
| Country FE | NO | YES | NO | YES |
| Lagged Dependent Variable | YES | NO | YES | NO |
| Usage Variables Lagged 1 Year | YES | YES | YES | YES |
| Standard errors in parentheses * p<0.05, ** p<0.01, *** p<0.001 | | | | |

| Table S4: Simultaneous effects of lagged antibiotic usage on animal and human resistance for individual bacteria. | | | | |
| --- | --- | --- | --- | --- |
| *For Salmonella* | | | | |
|  | (1) | (2) | (3) | (4) |
| Variables | ln (Animal Resistance) | | ln (Human Resistance) | |
| ln (Animal Usage) | 0.237***  (0.0305) | 0.441***  (0.0298) | 0.183***  (0.0339) | 0.534***  (0.0294) |
| ln (Human Usage) | 0.0732***  (0.0137) | 0.146***  (0.0138) | 0.0813***  (0.0129) | 0.140***  (0.0123) |
| Constant | 1.523  (0.887) | 65.45  (46.34) | 2.990***  (0.683) | 67.90  (47.54) |
| Observations | 960 | 1288 | 599 | 737 |
| R-squared | 0.541 | 0.366 | 0.637 | 0.495 |
| *For Campylobacter* | | | | |
| ln (Animal Usage) | 0.261***  (0.0632) | 0.427***  (0.0649) | -0.0565  (0.0494) | 0.394***  (0.116) |
| ln (Human Usage) | 0.0375  (0.0356) | 0.0916**  (0.0322) | -0.0330  (0.0317) | 0.187***  (0.0513) |
| Constant | -0.747  (1.885) | 57.77  (110.1) | -1.876  (1.577) | -71.80  (186.3) |
| Observations | 282 | 412 | 182 | 232 |
| R-squared | 0.610 | 0.408 | 0.838 | 0.212 |
| *For Escherichia* | | | | |
| ln (Animal Usage) | 0.137***  (0.0210) | 0.342***  (0.0288) | 0.0139  (0.00735) | 0.166***  (0.0159) |
| ln (Human Usage) | 0.0644***  (0.0106) | 0.184***  (0.0122) | 0.0218***  (0.00344) | 0.195***  (0.00407) |
| Constant | 1.273*  (0.583) | -13.56  (46.35) | 0.542**  (0.167) | -12.61  (21.51) |
| Observations | 1083 | 1346 | 496 | 603 |
| R-squared | 0.746 | 0.415 | 0.974 | 0.895 |
| Year FE | YES | YES | YES | YES |
| Country FE | NO | YES | NO | YES |
| Lagged Dependent Variable | YES | NO | YES | NO |
| Usage Variables Lagged 1 Year | YES | YES | YES | YES |
| Standard errors in parentheses * p<0.05, ** p<0.01, *** p<0.001 | | | | |

| Table S5: Effects of antibiotic usage on animal and human resistance, including additional covariates (Gross Domestic Product, Health Expenditure per capita and Corruption Perception Index). | | | | |
| --- | --- | --- | --- | --- |
| *For Combined Usage* | | | | |
|  | (1) | (2) | (3) | (4) |
| Variables | ln (Animal Resistance) | | ln (Human Resistance) | |
| ln (Combined Usage) | 0.145***  (0.0123) | 0.297***  (0.0111) | 0.0472***  (0.0877) | 0.295***  (0.0103) |
| Constant | 3.695***  (0.951) | 56.93  (36.86) | 1.422*  (0.674) | 50.86  (36.13) |
| Observations | 2528 | 2993 | 1211 | 1347 |
| R-squared | 0.503 | 0.257 | 0.790 | 0.362 |
| *For Simultaneous Usage* | | | | |
| ln (Animal Usage) | 0.191***  (0.0197) | 0.418***  (0.0235) | 0.0295*  (0.0143) | 0.369***  (0.0280) |
| ln (Human Usage) | 0.0762***  (0.00960) | 0.147***  (0.0104) | 0.0345***  (0.00686) | 0.165***  (0.0107) |
| Constant | 4.214***  (0.947) | 51.08  (36.80) | 1.608*  (0.680) | 52.48  (37.52) |
| Observations | 2528 | 2993 | 1211 | 1347 |
| R-squared | 0.505 | 0.252 | 0.789 | 0.351 |
| Year FE | YES | YES | YES | YES |
| Country FE | NO | YES | NO | YES |
| Lagged Dependent Variable | YES | NO | YES | NO |
| Additional Controls | YES | YES | YES | YES |
| Standard errors in parentheses * p<0.05, ** p<0.01, *** p<0.001 | | | | |

| Table S6: Effects of antibiotic usage on animal and human resistance, excluding countries with a population less than 6 million people. | | | | |
| --- | --- | --- | --- | --- |
| *For Combined Usage* | | | | |
|  | (1) | (2) | (3) | (4) |
| Variables | ln (Animal Resistance) | | ln (Human Resistance) | |
| ln (Combined Usage) | 0.139***  (0.0134) | 0.310***  (0.0122) | 0.0566***  (0.0115) | 0.334***  (0.0107) |
| Constant | 2.358**  (0.766) | -108.3**  (35.34) | 1.636*  (0.640) | -91.57  (49.37) |
| Observations | 2032 | 2594 | 929 | 1094 |
| R-squared | 0.539 | 0.269 | 0.804 | 0.401 |
| *For Simultaneous Usage* | | | | |
| ln (Animal Usage) | 0.179***  (0.0191) | 0.382***  (0.0213) | 0.0439**  (0.0144) | 0.376***  (0.0252) |
| ln (Human Usage) | 0.0558***  (0.00921) | 0.122***  (0.0101) | 0.0291***  (0.00737) | 0.144***  (0.0101) |
| Constant | 2.923***  (0.780) | -125.5***  (35.01) | 1.724**  (0.642) | -109.0*  (48.39) |
| Observations | 2032 | 2594 | 929 | 1094 |
| R-squared | 0.543 | 0.269 | 0.803 | 0.391 |
| Year FE | YES | YES | YES | YES |
| Country FE | NO | YES | NO | YES |
| Lagged Dependent Variable | YES | NO | YES | NO |
| Standard errors in parentheses * p<0.05, ** p<0.01, *** p<0.001 | | | | |

## Supplementary Figures

**Figure S1:** Frequency distribution of Usage in Tonnes and Resistance in % for food-producing animals and humans.

**Figure S2:** Frequency distribution of log transformed Usage in Tonnes and Resistance in % for food-producing animals and humans.


**Figure S3:** Box plots using raw values of usage and resistance variables shows the variables vary widely.

**Figure S4:** Showing log normalized box plots for Usage and Resistance Variables.
