# Supplementary figures and images for: The effect of antibiotic usage on resistance in humans and food-producing animals: a longitudinal, One Health analysis using European data
Source: Front Public Health. 2023 Jun 15;11:1170426. doi: 10.3389/fpubh.2023.1170426 (PMC10311110; doi:10.3389/fpubh.2023.1170426)

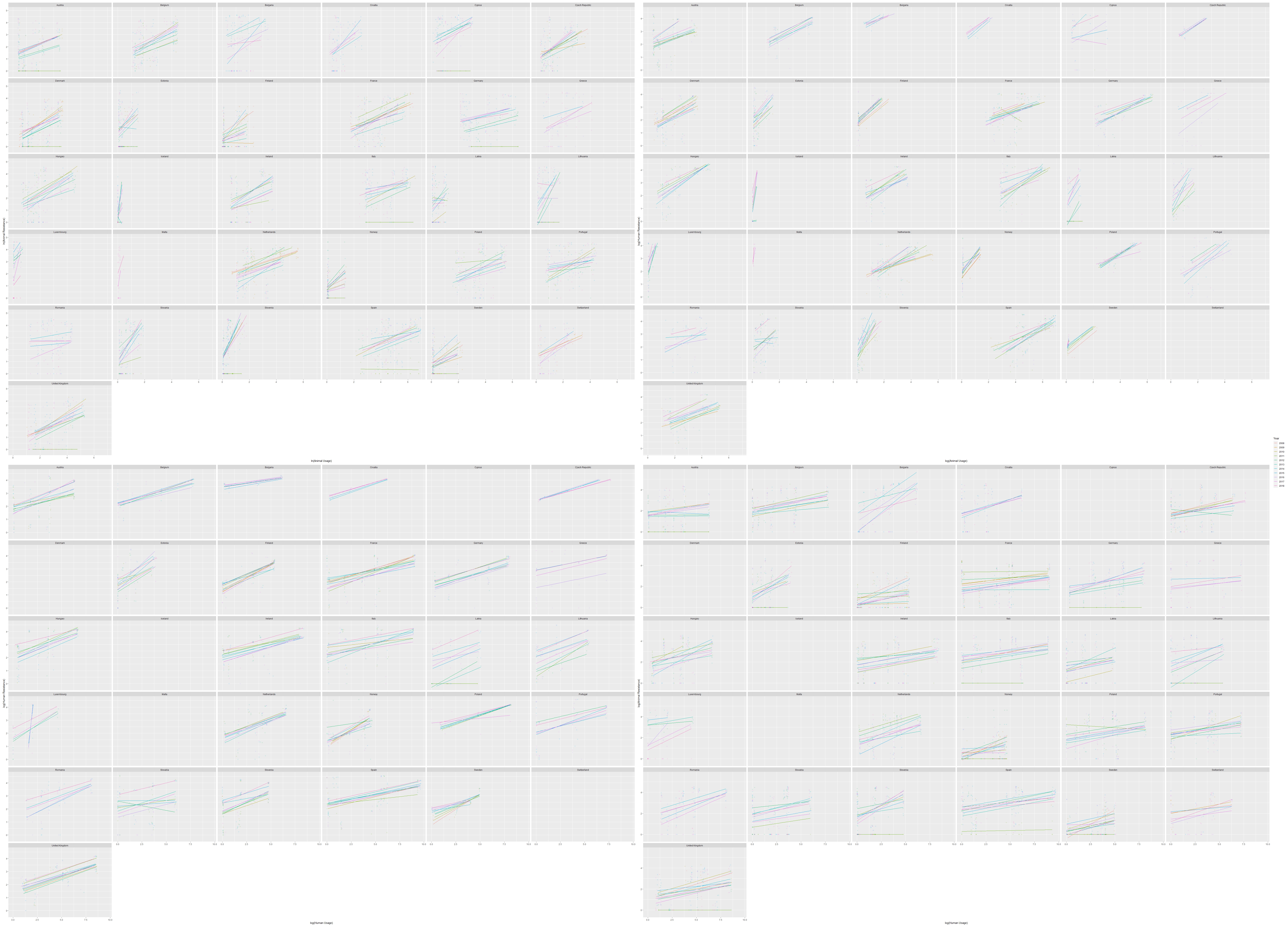

Supplement: Supplementary file 2 [file Image_1.JPEG]

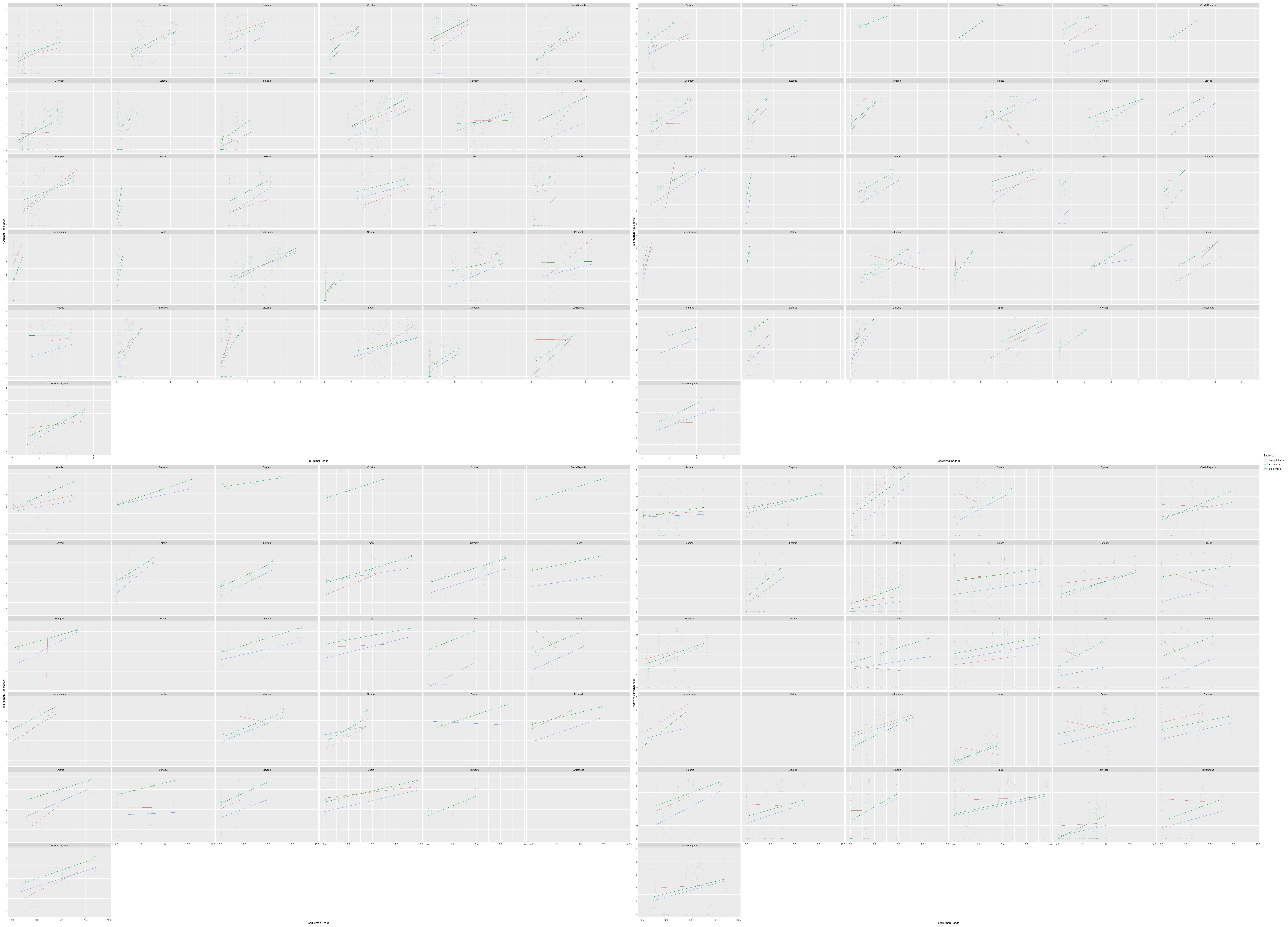

Supplement: Supplementary file 3 [file Image_2.JPEG]

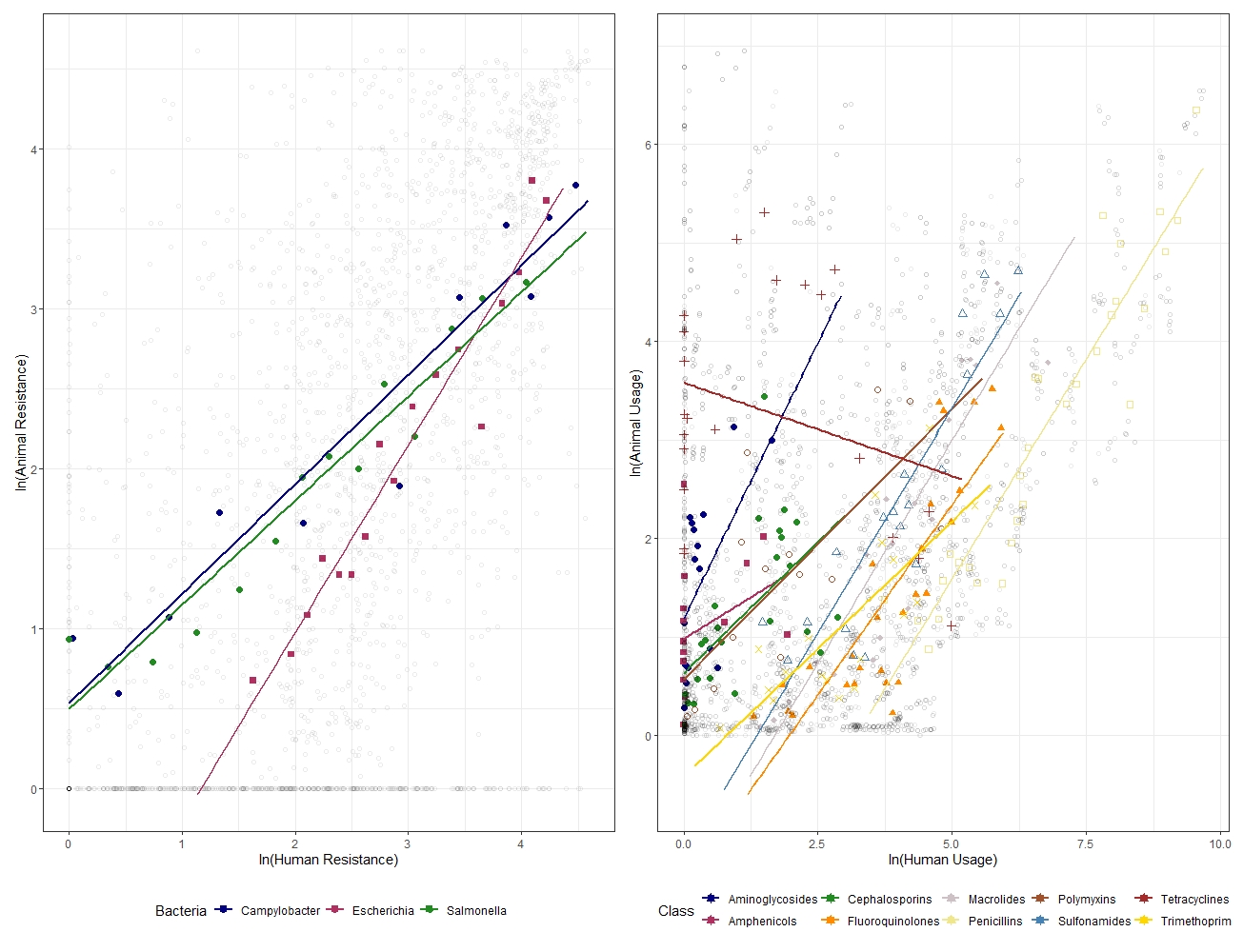

Supplement: Supplementary file 4 [file Image_3.JPEG]
